# Resolving the Controversy in Biexciton Binding Energy of Cesium Lead Halide Perovskite Nanocrystals through Heralded Single-Particle Spectroscopy

**DOI:** 10.1021/acsnano.1c06624

**Published:** 2021-11-30

**Authors:** Gur Lubin, Gili Yaniv, Miri Kazes, Arin Can Ulku, Ivan Michel Antolovic, Samuel Burri, Claudio Bruschini, Edoardo Charbon, Venkata Jayasurya Yallapragada, Dan Oron

**Affiliations:** † Department of Physics of Complex Systems, Weizmann Institute of Science, Rehovot 7610001, Israel; ‡ Department of Molecular Chemistry and Materials Science, Weizmann Institute of Science, Rehovot 7610001, Israel; § School of Engineering, École polytechnique fédérale de Lausanne (EPFL), Neuchâtel 2002, Switzerland; ∥ Department of Physics, Indian Institute of Technology Kanpur, Kanpur 208016, India

**Keywords:** perovskite nanocrystals, quantum dots, biexciton binding energy, single-particle spectroscopy, SPAD arrays

## Abstract

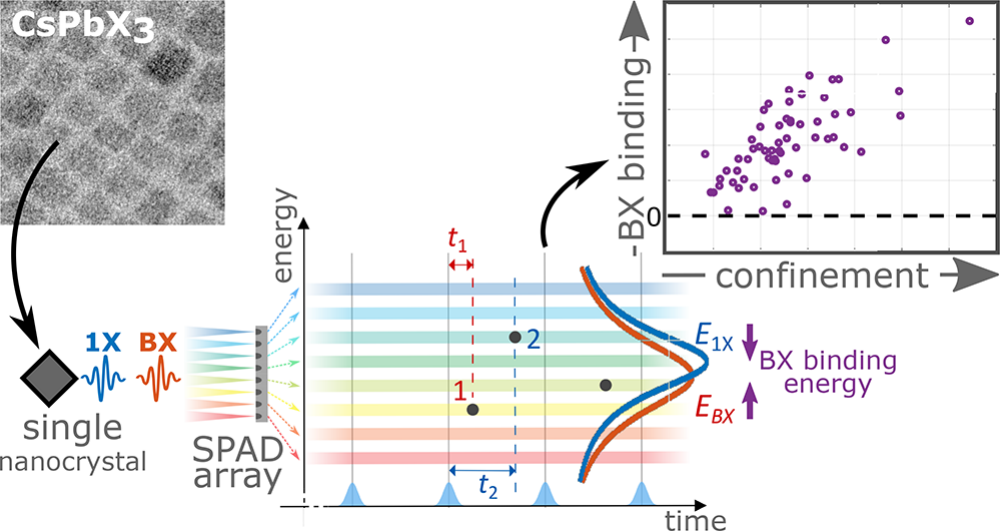

Understanding exciton−exciton interaction in multiply excited nanocrystals is crucial to their utilization as functional materials. Yet, for lead halide perovskite nanocrystals, which are promising candidates for nanocrystal-based technologies, numerous contradicting values have been reported for the strength and sign of their exciton−exciton interaction. In this work, we unambiguously determine the biexciton binding energy in single cesium lead halide perovskite nanocrystals at room temperature. This is enabled by the recently introduced single-photon avalanche diode array spectrometer, capable of temporally isolating biexciton−exciton emission cascades while retaining spectral resolution. We demonstrate that CsPbBr_3_ nanocrystals feature an attractive exciton−exciton interaction, with a mean biexciton binding energy of 10 meV. For CsPbI_3_ nanocrystals, we observe a mean biexciton binding energy that is close to zero, and individual nanocrystals show either weakly attractive or weakly repulsive exciton−exciton interaction. We further show that, within ensembles of both materials, single-nanocrystal biexciton binding energies are correlated with the degree of charge-carrier confinement.

Colloidal semiconductor nanocrystals (NCs) have been extensively studied over the last three decades, owing to the ease of their synthesis and tunability of their photophysical properties.^1^ Absorption of a photon by an NC leads to the formation of an exciton, a bound electron-hole pair, whose energy can be precisely tuned by varying the physical dimensions of the NC.^2^ In well passivated direct band gap NCs, the dominant relaxation route of excitons is *via* photoluminescence (PL). Additional complexity is introduced when NCs absorb multiple photons, generating multiexcitonic states. In the simplest of these states, the biexciton (BX), two excitons are confined within the NC.

PL from the BX state can serve as a probe to investigate exciton−exciton interaction within the NC. Relaxation from the BX to the singly excited (1X) state can occur through the radiative PL process or *via* nonradiative Auger processes.^3^ Hence, the probability of radiative relaxation from the BX state, the BX quantum yield, is indicative of the relative rates of the two processes. A cascaded radiative relaxation from BX to 1X and further to the ground (G) state results in the emission of two photons in rapid succession. The energy of the first photon (*E*_BX_) will be shifted from the second (*E*_1X_), according to exciton−exciton interaction. The value of this shift, the BX binding energy (ε_b_ ≡ *E*_1X_ – *E*_BX_), is defined to be positive for attractive interaction. In intrinsic homogeneous or type-I NCs, where all charge carriers are confined to the same volume, this interaction is typically attractive and stronger than in the bulk, due to the correlation energy of the confined excitons. In type-II heterostructure NCs, where electrons and holes are spatially separated, Coulombic repulsion of like-charged carriers can overwhelm this correlation energy and result in an overall repulsive interaction.^4^ Significant effort has been directed at the evaluation and control of this value in II-VI and III-V semiconductor NCs, as it is critical to enhance their performance in various applications, such as sources of quantum light,^5^ lasing media and LEDs,^3^ and photovoltaics.^6^

In recent years, there has been a surge of interest in lead halide perovskite (LHP) NCs of the form APbX_3_, where A is a monovalent cation and X a halide anion. Their prominent features, near unity PL quantum yield, defect tolerance, and tunable emission across the visible spectrum, have made them a promising candidate for various optoelectronic applications.^7,8^ Additionally, at cryogenic temperatures, they exhibit long PL coherence times, which are desirable for emerging quantum optical technologies such as generation of coherent single photons^9^ and entangled photon pairs.^10^ As in their II-VI and III-V counterparts, many of these applications stand to benefit from, or even depend on, reliable estimation of the BX binding energy.

However, the value of the BX binding energy in LHP NCs is a subject of current debate. Reported values for the prototypical all-inorganic CsPbBr_3_ NCs span the range between strongly attractive to strongly repulsive interaction (ε_b_ ≈ +100 meV^11^ to −100 meV;^12^ see Supporting Information Table S1). Common to all previous experimental works is their reliance on ensemble measurements. While these techniques proved invaluable in studying multiexcitonic states in NCs, their ensemble nature introduces several possible sources for the estimation errors which may underlie the existing discrepancies. First, ensemble methods require fitting data to a model, and quantitative results often depend on the model chosen to analyze and interpret the data.^13,14^ In particular, the BX contribution might be hard to disentangle from other photophysical or chemical processes such as charging or sintering,^15,16^ leading to ambiguities. Additionally, most methods require resolving the BX and 1X peaks spectrally, which might prove challenging when ε_b_ is much smaller than the 1X homogeneous and inhomogeneous spectral broadening.^15,16^ Finally, the size heterogeneity, inherent to colloidally synthesized NC ensembles, can introduce systematic biases due to the size dependent absorption cross section of the 1X and BX states.

Room temperature single-particle heralded spectroscopy has been recently introduced as a way to overcome these shortcomings of ensemble approaches.^16,17^ This is achieved by temporally isolating photon pairs originating from the BX→1X→G cascade of single particles and is hence free of all the aforementioned biases and ambiguities. In this work, we utilize this technique to unambiguously determine the BX binding energies of the prototypical LHP NCs CsPbBr_3_ and CsPbI_3_. All CsPbBr_3_ single particles measured featured an attractive exciton−exciton interaction (ε_b_ = 10 ± 6 meV), and a clear correlation of the BX binding energy with charge-carrier confinement was observed. Interestingly, CsPbI_3_ NCs showed either weakly attractive or weakly repulsive exciton−exciton interaction with an average around zero binding energy (ε_b_ = 1 ± 9 meV).

## Results

### Nanocrystals in This Work

**Figure 1:**
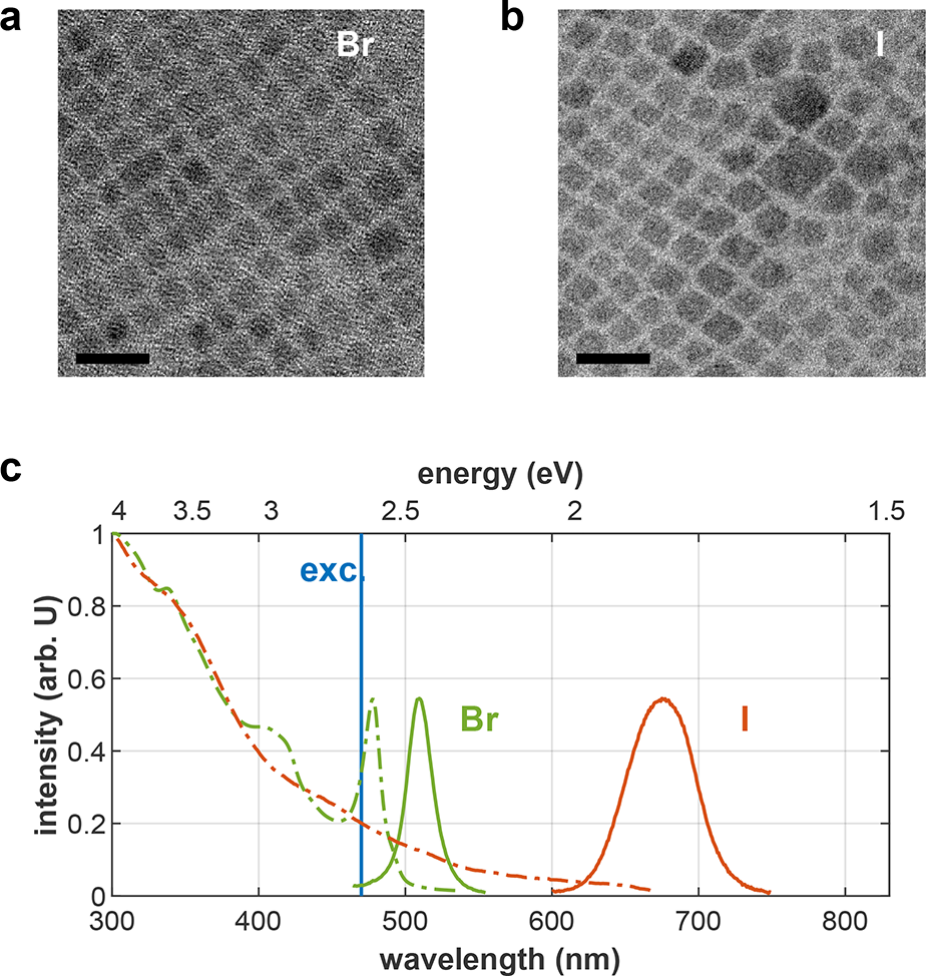
Particles investigated in this work. (a) Transmission electron micrograph of the CsPbBr_3_ NCs investigated in this work. (b) Transmission electron micrograph of the CsPbI_3_ NCs investigated in this work. Both scale bars are 20 nm. (c) Ensemble emission (solid lines) and absorption (dashed dotted lines) of the CsPbBr_3_ (green) and CsPbI_3_ (red) NCs. Blue line marks the excitation wavelength (470 nm).

Perovskite NCs investigated in this work were synthesized according to refs (18) and (19) (CsPbBr_3_), and ref (20) (CsPbI_3_), with minor modifications (see Methods section and Supporting Information section S2). CsPbBr_3_ NCs, seen in Figure 1a, feature an edge size distribution of 5.9 ± 1.3 nm, 2.44 eV ensemble emission peak, and ∼100% quantum yield. For CsPbI_3_, seen in Figure 1b, two size populations are visible. Smaller NCs (∼80% of the particles) with an edge size distribution of 7.2 ± 1.9 nm, and larger NCs with an edge size distribution of 15.4 ± 3.3 nm. The ensemble emission peak is at 1.84 eV, and the quantum yield is ∼42%. Samples of isolated nanocrystals were prepared by spin coating a dilute solution of the NCs dispersed in a 3 wt % solution of poly(methyl methacrylate) (PMMA) in toluene on a glass coverslip.

### Single-Particle Heralded Spectroscopy

**Figure 2:**
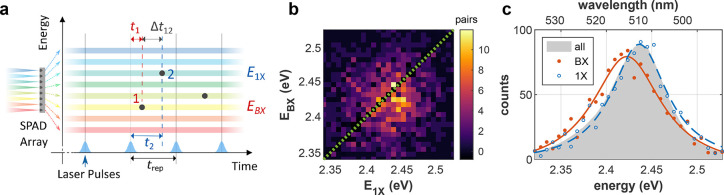
Heralded spectroscopy of a single particle. (a) A schematic illustration of the heralded spectroscopy scheme. A linear SPAD array is placed at the output of a grating spectrometer such that each SPAD pixel detects a different wavelength. The data from each SPAD pixel consists of the absolute arrival times of photons. By identifying the first and second arriving photons in each coincidence detection (BX and 1X, respectively), their corresponding energies can be extracted (*E*_BX_ and *E*_1X_). (b) 2D histogram of photon pairs following the same excitation pulse, from a 5 min measurement of a single CsPbBr_3_ NC. Green dashed line is a guide to the eye marking both photons with the same energy (undetectable by the system). (c) BX spectrum (red dots) and 1X spectrum (blue circles) extracted by full horizontal and full vertical binning of panel (b), respectively. Gray area is the 1X spectrum (normalized) extracted by summing over all detected photons. Red solid line and blue dashed line represent fits of the BX and 1X spectra, respectively, to Cauchy–Lorentz distributions. BX binding energy for this specific NC, estimated as the difference between the spectral peaks of the two fits, is ε_b_ = 13.5 ± 1.8 meV.

In order to measure the BX binding energy in single NCs, we use heralded spectroscopy, a technique that utilizes the temporal correlation of photon detections to unambiguously resolve the BX and 1X emission spectra. This technique was recently introduced and utilized to measure the BX binding energy of single CdSe/CdS/ZnS quantum dots at room temperature.^16^ Briefly, an inverted microscope with a high numerical aperture objective is used to focus pulsed laser illumination on a single NC, and collect the emitted fluorescence. The collected fluorescence is then passed through a Czerny–Turner spectrometer with a single-photon avalanche diode (SPAD) array detector, so that each detected photon is time-stamped according to its arrival time, and energy-stamped according to the array pixel it was detected in (Figure 2a). Post-selecting only photon pairs that follow the same excitation pulse robustly isolates BX-1X emission cascades from emission of other overlapping emitting states, such as 1X or trions. The pump power is adjusted so that the average number of photons absorbed by an NC per pump pulse (⟨*N*⟩) is low (<0.4; see Supporting Information section S3). This helps prevent rapid deterioration of the NCs and minimize excitation of higher multiexcitonic states. A thorough description of the system and technique is given in ref (16), and some modifications made to accommodate the different fluorescence parameters of the LHP NCs are described in the Methods section and in Supporting Information section S4.

Figure 2b is a 2D histogram of such post-selected photon pairs from a 5 min measurement of a single CsPbBr_3_ NC. It shows the energy of the first arriving photon (*E*_BX_) as a function of the second arriving photon (*E*_1X_) of the pair. The green dashed line is a guide to the eye, marking the same energy for both photons (undetectable by the system due to pixel dead time). The asymmetry of the histogram around this diagonal is indicative of an attractive exciton−exciton interaction (*E*_BX_ is typically smaller than *E*_1X_). This energy difference is quantified in Figure 2c where the BX (red dots) and 1X (blue circles) spectra are extracted by full horizontal and full vertical binning, respectively, of Figure 2b. This identification is corroborated by the good agreement between the 1X spectrum, and the spectrum of all detected photons (gray area, normalized). The emission peaks of the BX and 1X spectra are estimated from fits to Cauchy–Lorentz distributions (matching color lines), and the BX binding energy is estimated as the difference in peak positions. For this specific NC, ε_b_ = 13.5 ± 1.8 meV (all errors in this paper are estimated as the 68% confidence interval of the fit).

Two further insights were extracted from the same data set. First, the normalized second order correlation of photon arrival times (*g*^(2)^(0)) was calculated by the method described in ref (21). This value is defined as the ratio between the number of detection pairs following the same excitation pulse and the expected number for a classical Poissonian emitter. The presence of the additional exciton in a doubly excited NC increases the probability of nonradiative BX to 1X decay *via* Auger recombination. As a consequence, fewer photon pairs are emitted, leading to *g*^(2)^(0) < 1, a phenomenon termed photon antibunching. Hence, the value of *g*^(2)^(0) helps quantify the PL quantum yield of the BX state.^22^

Second, the *time-gated* second order correlation of photon arrival times (*ĝ*^(2)^(0)) was calculated. This is performed by post-selecting only detections arriving within a time window of 1–30 ns after any excitation pulse, and applying the same *g*^(2)^(0) calculation procedure to the resulting filtered data. Most multiexciton emission processes occur at time scales shorter than 1 ns (see Supporting Information section S5), and are therefore, filtered out by this time window. In single NCs, multiexciton states are the only source for multiple photon detections following the same excitation pulse. Therefore, a low *ĝ*^(2)^(0) is a good indication of whether the observed emission originates from a single particle or not^23,24^ (*g*^(2)^(0) and *ĝ*^(2)^(0) are further discussed in Supporting Information section S3). For this specific NC, *g*^(2)^(0) = 0.175 ± 0.008 and *ĝ*^(2)^(0) = 0.012 ± 0.003.

### CsPbBr_3_ NCs

**Figure 3:**
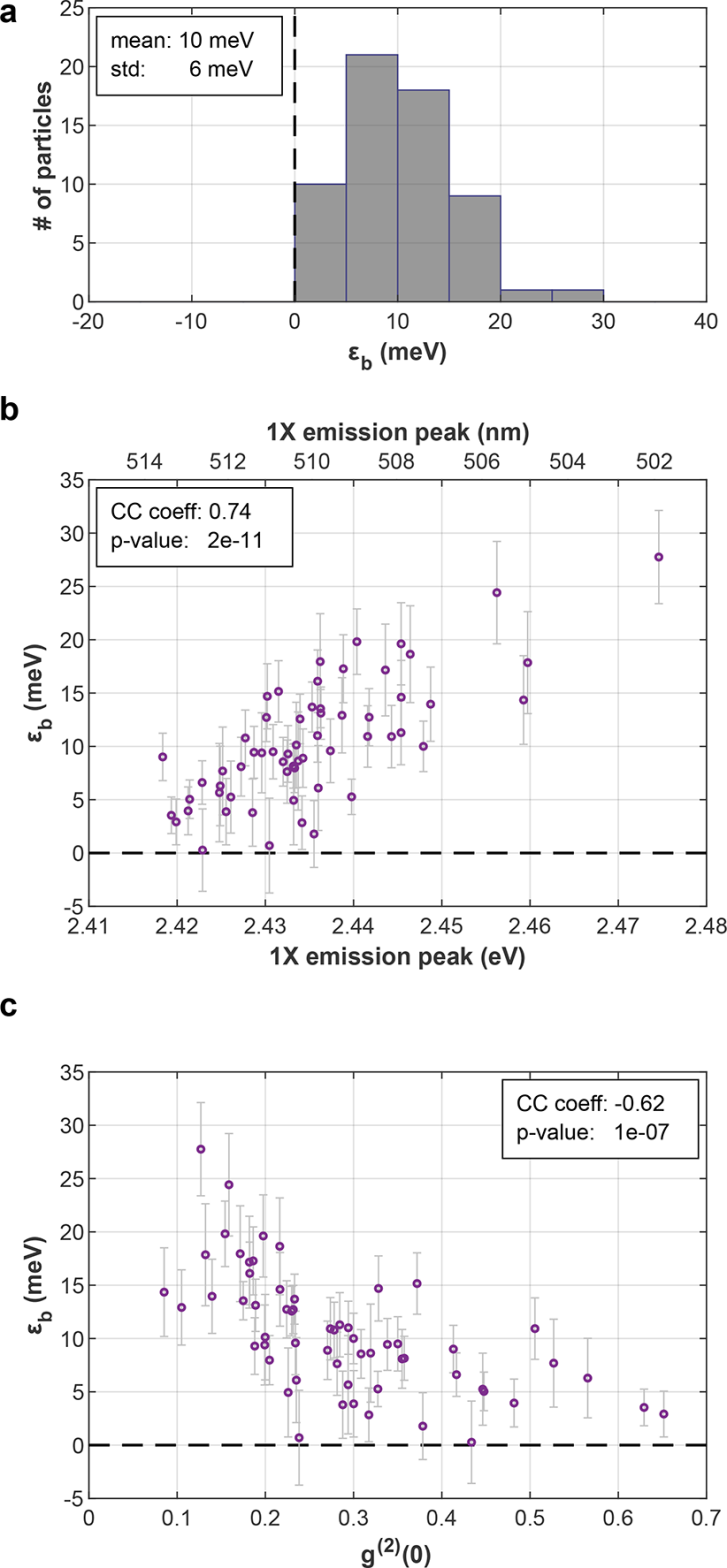
CsPbBr_3_ biexciton binding energy. (a) BX binding energy histogram for 60 NCs. Mean single-particle error is ±3.1 meV. (b) BX binding energy as a function of 1X emission peak. (c) BX binding energy as a function of *g*^(2)^(0). std: standard deviation; CC coeff: cross-correlation coefficient; *p*-value: *p*-value of the cross-correlation.

Figure 3a shows the BX binding energy of 60 single CsPbBr_3_ NCs, determined using the procedure illustrated in Figure 2. In our measurements, we maintain ⟨*N*⟩ ∼ 0.1 and obtain a mean single-particle ε_b_ error of ±3.1 meV. To filter out accidental measurements of non-isolated NCs, only measurements where *ĝ*^(2)^(0) < 0.2 are considered. All particles feature an attractive exciton−exciton interaction (ε_b_ > 0), and the distribution is centered around ε_b_ = 10 ± 6 meV. Figure 3b displays the binding energy of each NC as a function of the 1X emission peak position. A clear correlation between the two values is evident. This can be interpreted as the effect stronger charge-carrier confinement has on both the 1X emission peak (stronger confinement is correlated with higher energy emission peak) and the binding energy (stronger confinement is correlated with stronger interaction of the two excitons). The trend and magnitude are in reasonable agreement with theoretical predictions recently made by Nguyen *et al.*,^25^ and bounds suggested by Shulenberger *et al.*(15)

The suggested interpretation is further corroborated in Figure 3c. Here, the BX binding energy is plotted as a function of *g*^(2)^(0), another value indicative of charge-carrier confinement. Namely, tighter confinement increases the rate of Auger processes^3^ and, consequently, reduces the yield of the competing radiative BX decay process, evident in lower *g*^(2)^(0). Therefore, the inverse correlation of ε_b_ with *g*^(2)^(0) evident in Figure 3c can be seen as pointing to the same underlying correlation of the BX binding energy with charge-carrier confinement.

### CsPbI_3_ NCs

**Figure 4:**
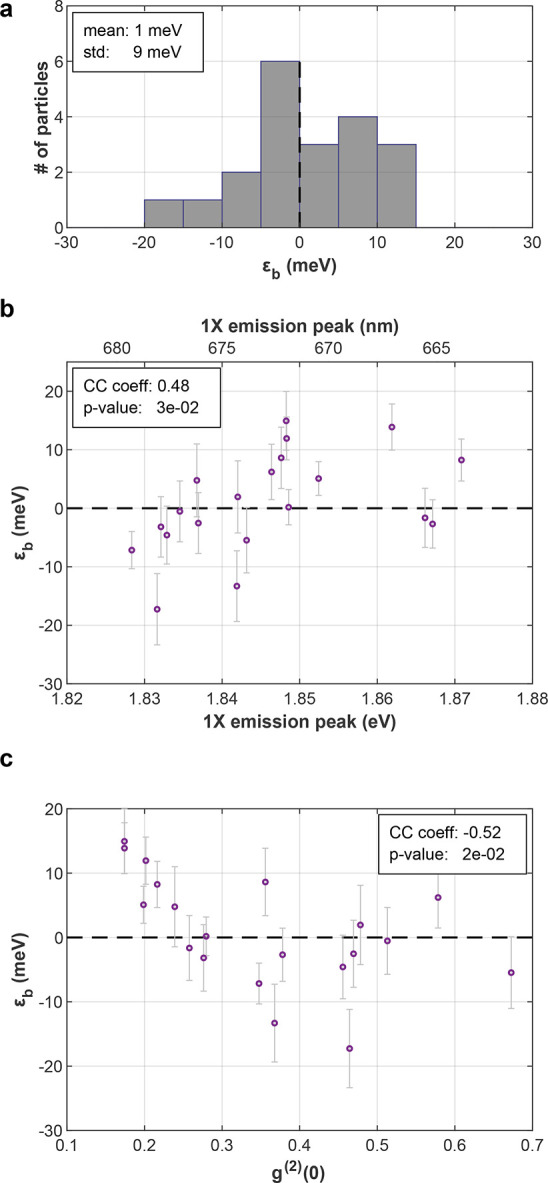
CsPbI_3_ biexciton binding energy. (a) BX binding energy histogram for 20 NCs. Mean single-particle error is ±4.8 meV. (b) BX binding energy as a function of 1X emission peak. (c) BX binding energy as a function of *g*^(2)^(0). std: standard deviation; CC coeff: cross-correlation coefficient; *p*-value: *p*-value of the cross-correlation.

CsPbI_3_ NCs BX binding energies were measured by the same technique (Figure 4). Results feature ε_b_ values distributed around zero (ε_b_ = 1 ± 9 meV, ⟨*N*⟩ ∼ 0.3, mean single-particle ε_b_ error: ±4.8 meV). The trends observed for CsPbBr_3_ are visible here as well, where higher 1X emission peak energy and lower *g*^(2)^(0), or stronger confinement, are correlated with stronger attractive interaction (Figure 4b,c). As a result, while ε_b_ values are mostly within reasonable error from zero, NCs featuring a 1X emission peak lower (higher) than 1.845 eV or *g*^(2)^(0) higher (lower) than 0.25 typically display a small negative (positive) ε_b_ value. While the results are not as conclusive as for CsPbBr_3_, they suggest that the weak exciton−exciton interaction in CsPbI_3_ NCs can be either repulsive or attractive, depending on the exact parameters of the single particle.

It is noteworthy that CsPbI_3_ measurements were significantly more challenging than their CsPbBr_3_ counterparts. This is due to two factors. First, CsPbI_3_ NCs synthesized were typically less emissive and less stable under the conditions of our measurements. That resulted in many NCs deteriorating during the measurement (PL declines to near zero), before enough photon pairs were detected to extract reliable spectra fits. Second, current SPAD array technology is less sensitive at these longer wavelengths.^26^ The SPAD array detector used in this work has roughly twice the photon detection efficiency at the CsPbBr_3_ emission peak compared to the CsPbI_3_ emission peak. These two factors resulted in the smaller statistics and larger errors for CsPbI_3_ NCs in this work.

## Discussion

The BX binding energies presented in this paper are at the lower range of values previously reported in the literature for these NCs (see a table of previously reported values in Supporting Information section S1). While in some cases this might be attributed to the potential pitfalls associated with ensemble measurements discussed in the introduction, it is also important to consider the possibility that heralded spectroscopy and ensemble measurements probe the NC in a qualitatively different excitation state. For example, one widely adopted ensemble technique for estimating BX binding energy involves recording the transient absorption (TA) spectrum of a probe pulse at very short (<1 ps) delays from a pump pulse, that is, before the relaxation of hot carriers to the band edge.^13,27,28^ The hot carrier pair generated by the pump shifts the spectral position of the absorption resonance for the probe photon, and this shift is recorded as the exciton−exciton interaction energy. In contrast, results presented in this paper rely on measurements of photon pairs emitted from individual BX→1X→G cascades following the excitation pulse. Since the PL decay lifetimes of the BX and 1X states are significantly longer than the time scales of hot carrier relaxation in the NCs (see Supporting Information section S5), our measurements probe the NC only after the hot carrier pairs have relaxed to the band edge.

Since the wave function of the hot exciton differs from that of a band-edge 1X state, the interaction energies may be different in the two cases. Studies on PbS nanocrystals indicate that the magnitude of interaction between a hot exciton and a band-edge exciton is larger than between two band-edge excitons.^29^ For CsPbI_3_ NCs, a recent study indicates that the estimated ε_b_ increases as the pump wavelength decreases in short-delay TA experiments.^30^ Similar trends have been demonstrated for CsPbBr_3_ at cryogenic temperatures using two-dimensional electron spectroscopy.^31^ In addition, analyses of TA measurements by Ashner *et al.*, that do not employ short-delay spectra, did not result in large positive values (but rather in small negative values of a few meV).^14^ Together, these observations suggest that ε_b_ measured when both excitons are at the band edge would be lower than that measured when the first exciton is still hot. In this sense, heralded spectroscopy and short-delay TA are complementary measurements of band-edge and hot exciton−exciton interaction, respectively, and a careful comparison of the two can help uncover insights into dynamics of exciton interactions in NCs.

Negative ε_b_ values, observed only for CsPbI_3_ NCs in this work, are less often reported in the literature for similar NCs (see Supporting Information section S1). The origin of this repulsive interaction is not immediately apparent from existing theoretical models of intrinsic homogeneous semiconductor NCs.^25,32^ One possible explanation is a modification of the charge-carrier wave functions induced by surface ligands.^33^ This can result in a type-II potential landscape, where the electrons or the holes are localized at the NC surface, and Coulomb repulsion might dominate the exciton−exciton interaction. Alternatively, the electrostatic field generated by charge carriers trapped in the ligand-induced trap states can result in charge separation, and a similar repulsive interaction. Another possibility, suggested by Ashner *et al.*,^14^ is the formation of polarons, supported by the deformable nature of the perovskite lattice. The results presented in this work cannot pinpoint a certain mechanism, and present limited statistics of negative ε_b_ values. However, the apparent observation of a repulsive exciton−exciton interaction in a homogeneous nanocrystal highlights the importance of further investigating the effect of surface chemistry, environment, and perovskite lattice on charge-carrier interaction in LHP NCs.

In addition to the BX binding energy, the single-particle nature of our technique also allows the investigation of the BX spectral line width. As is evident from Figure 2c and Figure S5, the CsPbBr_3_ NCs BX spectrum is broader by a factor of 1.4 ± 0.4 compared to the 1X spectrum (measurements of CsPbI_3_ NCs suggest a similar factor, but at a lower confidence). A possible explanation for this broadening is the presence of multiple decay routes from the BX to the 1X state, possibly through thermally excited hot BX states which are close energetically to the lowest BX state.^31,34^ We note that this is qualitatively different from observations in core/shell/shell CdSe/CdS/ZnS NCs.^16,17^ However, II-VI semiconductor NCs feature more pronounced spectral diffusion compared to LHP NCs.^35,36^ As demonstrated in ref (16), attenuation of the BX spectral diffusion as compared to the 1X spectral diffusion can overwhelm BX broadening effects, if they exist, and lead to a BX spectrum that is narrower than the 1X.

## Conclusions

Heralded spectroscopy enables us to unambiguously determine the biexciton binding energy (ε_b_) of single lead halide perovskite nanocrystals. Using this technique, we demonstrate that ∼6 nm edge CsPbBr_3_ nanocrystals feature an attractive exciton−exciton interaction of ε_b_ = 10 ± 6 meV, which lies at the lower range of previously reported values. Interestingly, within the ensemble of ∼7 nm edge CsPbI_3_ nanocrystals, some exhibit weak attractive interactions, whereas others appear to exhibit weak exciton−exciton repulsion. This rarely observed phenomenon in homogeneous nanocrystals warrants further investigation of charge-carrier interactions in these particles. In nanocrystals of both materials, the strength of attractive interaction exhibits a clear correlation with single-exciton emission peak position and photoluminescence antibunching (*g*^(2)^(0)), highlighting the dependence of ε_b_ on charge-carrier confinement. These insights into the physics of exciton interactions in lead halide perovskite nanocrystals can enable the development of better engineered nanocrystals for future optoelectronic technologies. Moreover, the ability to determine the biexciton binding energy of single nanocrystals at room temperature is instrumental to their utilization in quantum technologies.

## Methods

### Synthesis of CsPbBr_3_ Nanocrystals

CsPbBr_3_ NCs were synthesized according to a reported recipe^18^ with slight modifications. ODE (5 mL) and PbBr_2_ (69 mg) were mixed and dried under vacuum for 1 h at 120  °C. Then, under an Ar atmosphere, dried OA (0.5 mL) and dried OLA (0.5 mL) were injected to the mixture and the temperature was raised to 180 °C and kept for 10 min. Then, Cs-oleate solution (0.4 mL) was swiftly injected, and after 25 s, the reaction mixture was cooled by an ice water bath. The NCs were purified from the crude solution by centrifugation and redispersed in anhydrous toluene. Following, surface treatment was performed by a saturated NH_4_BF_4_ toluene solution (1 mL) stirred together with 25 mL of CsPbBr_3_ NCs in toluene (following ref (19) with slight modifications).

### Synthesis of CsPbI_3_ Nanocrystals

CsPbI_3_ NCs were synthesized according to the recipe reported in ref (20) with minor modifications. ODE (5 mL), PbI_2_ (87 mg), OLA (1 mL, dry), TDPA (120 mg), and TOPO (1.5 mg) were mixed and dried under vacuum for 1 h at 120  °C. The temperature was raised to 280 °C and kept for 10 min under an Ar atmosphere. Then Cs-oleate solution (0.4 mL) was quickly injected, and after 15 s, the reaction mixture was cooled by an ice-water bath. The crude solution was purified by two-step centrifugation in hexane.

See further details of the synthesis protocols in Supporting Information section S2.

### Optical Setup

The SPAD array spectrometer is built around a commercial inverted microscope (Eclipse T*i*-U, Nikon). An oil immersion objective (×100, 1.3 NA, Nikon) is used to focus light from a pulsed laser source (470 nm, 5 MHz, LDH-P-C-470B, PicoQuant) on a single NC and collect the emitted PL. The emitted light is then filtered through a dichroic mirror (FF484-FDi02-t3, Semrock) and a long-pass filter (BLP01-473R, Semrock). The magnified image plane (×150) serves as the input for a Czerny–Turner spectrometer that consists of a 4-f system (AC254-300-A-ML and AC254-100-A-ML, Thorlabs) with a blazed grating (53-*-321R, Richardson) at the Fourier plane. At the output image plane of the spectrometer, a 512 pixel on-chip linear SPAD array is placed. In our experiments, 30–43 pixels of the array were used (see Supporting Information section S4). The physical pixel pitch is 52.4 μm, corresponding to ∼1.5 nm wavelength difference of impinging photons between neighboring pixels. Time tagging is done by an array of 64 time-to-digital converters (TDCs) implemented on a field-programmable gate array (FPGA). The temporal instrument response function of the system features a ∼180 ps full width at half maximum (FWHM).

See further details of the experimental setup in ref (16) and Supporting Information section S4.

## Supporting Information Available

List of previously reported biexciton binding energies; nanocrystal synthesis protocol; details of supporting analyses of ⟨*N*⟩, *g*^(2)^(0), and *ĝ*^(2)^(0); system parameters; photoluminescence decay lifetime estimation; biexciton spectrum broadening (PDF)

## References

[ref1] Efros A. L.; Brus L. E. Nanocrystal Quantum Dots: From Discovery to Modern Development. ACS Nano 2021, 15, 6192–6210. 10.1021/acsnano.1c01399. 33830732

[ref2] Brus L. E. Electron-Electron and Electron-Hole Interactions in Small Semiconductor Crystallites: The Size Dependence of the Lowest Excited Electronic State. J. Chem. Phys. 1984, 80, 4403–4409. 10.1063/1.447218.

[ref3] Melnychuk C.; Guyot-Sionnest P. Multicarrier Dynamics in Quantum Dots. Chem. Rev. 2021, 121, 2325–2372. 10.1021/acs.chemrev.0c00931. 33428388

[ref4] Oron D.; Kazes M.; Banin U. Multiexcitons in Type-II Colloidal Semiconductor Quantum Dots. Phys. Rev. B: Condens. Matter Mater. Phys. 2007, 75, 035330 10.1103/PhysRevB.75.035330.

[ref5] Senellart P.; Solomon G.; White A. High-Performance Semiconductor Quantum-Dot Single-Photon Sources. Nat. Nanotechnol. 2017, 12, 1026–1039. 10.1038/nnano.2017.218. 29109549

[ref6] Kramer I. J.; Sargent E. H. Colloidal Quantum Dot Photovoltaics: A Path Forward. ACS Nano 2011, 5, 8506–8514. 10.1021/nn203438u. 21967723

[ref7] Protesescu L.; Yakunin S.; Bodnarchuk M. I.; Krieg F.; Caputo R.; Hendon C. H.; Yang R. X.; Walsh A.; Kovalenko M. V. Nanocrystals of Cesium Lead Halide Perovskites (CsPbX3, X = Cl, Br, and I): Novel Optoelectronic Materials Showing Bright Emission with Wide Color Gamut. Nano Lett. 2015, 15, 3692–3696. 10.1021/nl5048779. 25633588 PMC4462997

[ref8] Kovalenko M. V.; Protesescu L.; Bodnarchuk M. I. Properties and Potential Optoelectronic Applications of Lead Halide Perovskite Nanocrystals. Science 2017, 358, 745–750. 10.1126/science.aam7093. 29123061

[ref9] Utzat H.; Sun W.; Kaplan A. E.; Krieg F.; Ginterseder M.; Spokoyny B.; Klein N. D.; Shulenberger K. E.; Perkinson C. F.; Kovalenko M. V.; Bawendi M. G. Coherent Single-Photon Emission from Colloidal Lead Halide Perovskite Quantum Dots. Science 2019, 363, 1068–1072. 10.1126/science.aau7392. 30792359

[ref10] Akopian N.; Lindner N. H.; Poem E.; Berlatzky Y.; Avron J.; Gershoni D.; Gerardot B. D.; Petroff P. M. Entangled Photon Pairs from Semiconductor Quantum Dots. Phys. Rev. Lett. 2006, 96, 130501 10.1103/PhysRevLett.96.130501. 16711973

[ref11] Castañeda J. A.; Nagamine G.; Yassitepe E.; Bonato L. G.; Voznyy O.; Hoogland S.; Nogueira A. F.; Sargent E. H.; Cruz C. H.; Padilha L. A. Efficient Biexciton Interaction in Perovskite Quantum Dots under Weak and Strong Confinement. ACS Nano 2016, 10, 8603–8609. 10.1021/acsnano.6b03908. 27574807

[ref12] Dana J.; Binyamin T.; Etgar L.; Ruhman S. Unusually Strong Biexciton Repulsion Detected in Quantum Confined CsPbBr3 Nanocrystals with Two and Three Pulse Femtosecond Spectroscopy. ACS Nano 2021, 15, 9039–9047. 10.1021/acsnano.1c02123. 33974397

[ref13] Makarov N. S.; Guo S.; Isaienko O.; Liu W.; Robel I.; Klimov V. I. Spectral and Dynamical Properties of Single Excitons, Biexcitons, and Trions in Cesium-Lead-Halide Perovskite Quantum Dots. Nano Lett. 2016, 16, 2349–2362. 10.1021/acs.nanolett.5b05077. 26882294

[ref14] Ashner M. N.; Shulenberger K. E.; Krieg F.; Powers E. R.; Kovalenko M. V.; Bawendi M. G.; Tisdale W. A. Size-Dependent Biexciton Spectrum in CsPbBr3 Perovskite Nanocrystals. ACS Energy Letters 2019, 4, 2639–2645. 10.1021/acsenergylett.9b02041.

[ref15] Shulenberger K. E.; Ashner M. N.; Ha S. K.; Krieg F.; Kovalenko M. V.; Tisdale W. A.; Bawendi M. G. Setting an Upper Bound to the Biexciton Binding Energy in CsPbBr3 Perovskite Nanocrystals. J. Phys. Chem. Lett. 2019, 10, 5680–5686. 10.1021/acs.jpclett.9b02015. 31502848

[ref16] Lubin G.; Tenne R.; Ulku A. C.; Antolovic I. M.; Burri S.; Karg S.; Yallapragada V. J.; Bruschini C.; Charbon E.; Oron D. Heralded Spectroscopy Reveals Exciton-Exciton Correlations in Single Colloidal Quantum Dots. Nano Lett. 2021, 21, 6756–6763. 10.1021/acs.nanolett.1c01291. 34398604 PMC8397400

[ref17] Vonk S. J. W.; Heemskerk B. A. J.; Keitel R. C.; Hinterding S. O. M.; Geuchies J. J.; Houtepen A. J.; Rabouw F. T. Biexciton Binding Energy and Line Width of Single Quantum Dots at Room Temperature. Nano Lett. 2021, 21, 5760–5766. 10.1021/acs.nanolett.1c01556. 34133188 PMC8283756

[ref18] Cao Y.; Zhu W.; Li L.; Zhang Z.; Chen Z.; Lin Y.; Zhu J. J. Size-Selected and Surface-Passivated CsPbBr3 Perovskite Nanocrystals for Self-Enhanced Electrochemiluminescence in Aqueous Media. Nanoscale 2020, 12, 7321–7329. 10.1039/D0NR00179A. 32202287

[ref19] Ahmed T.; Seth S.; Samanta A. Boosting the Photoluminescence of CsPbX3 (X = Cl, Br, I) Perovskite Nanocrystals Covering a Wide Wavelength Range by Postsynthetic Treatment with Tetrafluoroborate Salts. Chem. Mater. 2018, 30, 3633–3637. 10.1021/acs.chemmater.8b01235.

[ref20] Pan L.; Zhang L.; Qi Y.; Conkle K.; Han F.; Zhu X.; Box D.; Shahbazyan T. V.; Dai Q. Stable CsPbI3 Nanocrystals Modified by Tetra-*n*-Butylammonium Iodide for Light-Emitting Diodes. ACS Applied Nano Materials 2020, 3, 9260–9267. 10.1021/acsanm.0c01887.

[ref21] Lubin G.; Tenne R.; Antolovic I. M.; Charbon E.; Bruschini C.; Oron D. Quantum Correlation Measurement with Single Photon Avalanche Diode Arrays. Opt. Express 2019, 27, 32863–32882. 10.1364/OE.27.032863. 31878363

[ref22] Nair G.; Zhao J.; Bawendi M. G. Biexciton Quantum Yield of Single Semiconductor Nanocrystals from Photon Statistics. Nano Lett. 2011, 11, 1136–1140. 10.1021/nl104054t. 21288042 PMC3278281

[ref23] Mangum B. D.; Ghosh Y.; Hollingsworth J. A.; Htoon H. Disentangling the Effects of Clustering and Multi-Exciton Emission in Second-Order Photon Correlation Experiments. Opt. Express 2013, 21, 7419 10.1364/OE.21.007419. 23546125 PMC3635699

[ref24] Benjamin E.; Yallapragada V. J.; Amgar D.; Yang G.; Tenne R.; Oron D. Temperature Dependence of Excitonic and Biexcitonic Decay Rates in Colloidal Nanoplatelets by Time-Gated Photon Correlation. J. Phys. Chem. Lett. 2020, 11, 6513–6518. 10.1021/acs.jpclett.0c01628. 32693606 PMC7458474

[ref25] Nguyen T. P.; Blundell S. A.; Guet C. Calculation of the Biexciton Shift in Nanocrystals of Inorganic Perovskites. Phys. Rev. B: Condens. Matter Mater. Phys. 2020, 101, 125424 10.1103/PhysRevB.101.125424.

[ref26] Antolovic I. M.; Bruschini C.; Charbon E. Dynamic Range Extension for Photon Counting Arrays. Opt. Express 2018, 26, 22234 10.1364/OE.26.022234. 30130919

[ref27] Klimov V. I. Spectral and Dynamical Properties of Multiexcitons in Semiconductor Nanocrystals. Annu. Rev. Phys. Chem. 2007, 58, 635–673. 10.1146/annurev.physchem.58.032806.104537. 17163837

[ref28] Aneesh J.; Swarnkar A.; Kumar Ravi V.; Sharma R.; Nag A.; Adarsh K. V. Ultrafast Exciton Dynamics in Colloidal CsPbBr3 Perovskite Nanocrystals: Biexciton Effect and Auger Recombination. J. Phys. Chem. C 2017, 121, 4734–4739. 10.1021/acs.jpcc.7b00762.

[ref29] Geiregat P.; Houtepen A.; Justo Y.; Grozema F. C.; Van Thourhout D.; Hens Z. Coulomb Shifts upon Exciton Addition to Photoexcited PbS Colloidal Quantum Dots. J. Phys. Chem. C 2014, 118, 22284–22290. 10.1021/jp505530k.

[ref30] Yumoto G.; Tahara H.; Kawawaki T.; Saruyama M.; Sato R.; Teranishi T.; Kanemitsu Y. Hot Biexciton Effect on Optical Gain in CsPbI3 Perovskite Nanocrystals. J. Phys. Chem. Lett. 2018, 9, 2222–2228. 10.1021/acs.jpclett.8b01029. 29644864

[ref31] Huang X.; Chen L.; Zhang C.; Qin Z.; Yu B.; Wang X.; Xiao M. Inhomogeneous Biexciton Binding in Perovskite Semiconductor Nanocrystals Measured with Two-Dimensional Spectroscopy. J. Phys. Chem. Lett. 2020, 11, 10173–10181. 10.1021/acs.jpclett.0c03153. 33197186

[ref32] Hu Y. Z.; Koch S. W.; Lindberg M.; Peyghambarian N.; Pollock E. L.; Abraham F. F. Biexcitons in Semiconductor Quantum Dots. Phys. Rev. Lett. 1990, 64, 1805–1807. 10.1103/PhysRevLett.64.1805. 10041493

[ref33] Baker D. R.; Kamat P. V. Tuning the Emission of CdSe Quantum Dots by Controlled Trap Enhancement. Langmuir 2010, 26, 11272–11276. 10.1021/la100580g. 20373780

[ref34] Mueller S.; Lüttig J.; Brenneis L.; Oron D.; Brixner T. Observing Multiexciton Correlations in Colloidal Semiconductor Quantum Dots *via* Multiple-Quantum Two-Dimensional Fluorescence Spectroscopy. ACS Nano 2021, 15, 4647–4657. 10.1021/acsnano.0c09080. 33577282

[ref35] Rainò G.; Nedelcu G.; Protesescu L.; Bodnarchuk M. I.; Kovalenko M. V.; Mahrt R. F.; Stöferle T. Single Cesium Lead Halide Perovskite Nanocrystals at Low Temperature: Fast Single-Photon Emission, Reduced Blinking, and Exciton Fine Structure. ACS Nano 2016, 10, 2485–2490. 10.1021/acsnano.5b07328. 26771336 PMC4768330

[ref36] Hu F.; Yin C.; Zhang H.; Sun C.; Yu W. W.; Zhang C.; Wang X.; Zhang Y.; Xiao M. Slow Auger Recombination of Charged Excitons in Nonblinking Perovskite Nanocrystals without Spectral Diffusion. Nano Lett. 2016, 16, 6425–6430. 10.1021/acs.nanolett.6b02874. 27689439

