# Supplementary material for: Resolving the Controversy in Biexciton Binding Energy of Cesium Lead Halide Perovskite Nanocrystals through Heralded Single-Particle Spectroscopy
Source: ACS Nano. 2021 Nov 30;15(12):19581–7. doi: 10.1021/acsnano.1c06624 (PMC8717625; doi:10.1021/acsnano.1c06624)
Supplement: Supplementary file 1 — nn1c06624_si_001.pdf [file nn1c06624_si_001.pdf]

Resolving the Controversy in Biexciton Binding Energy of Cesium Lead Halide Perovskite Nanocrystals through Heralded Single-Particle Spectroscopy

Gur Lubin,† Gili Yaniv,‡ Miri Kazes,‡ Arin Can Ulku,¶ Ivan Michel Antolovic,¶ Samuel Burri,¶ Claudio Bruschini,¶ Edoardo Charbon,¶ Venkata Jayasurya Yallapragada,*,†,§ and Dan Oron*,‡

†*Deptartment of Physics of Complex Systems, Weizmann Institute of Science, Rehovot 7610001, Israel*
‡*Deptartment of Molecular Chemistry and Materials Science, Weizmann Institute of Science, Rehovot 7610001, Israel*
¶*School of Engineering, École polytechnique fédérale de Lausanne (EPFL), Neuchâtel 2002, Switzerland*
§*Department of Physics, Indian Institute of Technology Kanpur, Kanpur 208016, India*

E-mail: jayasurya@iitk.ac.in; dan.oron@weizmann.ac.il

Abstract

This supporting information describes in greater detail the synthesis, data analysis and system parameters, as well as provides some additional information to the work described in "Resolving the controversy in biexciton binding energy of cesium lead halide perovskite nanocrystals through heralded single-particle spectroscopy". Sections are brought in the order of their reference in the main text: list of previously reported biexciton binding energies; nanocrystal synthesis protocol; details of supporting analyses $\langle N \rangle$, $g^{(2)}(0)$ and $\hat{g^{(2)}}(0)$; system parameters; photoluminescence decay lifetime estimation; biexciton spectrum broadening.

S1 Published Values of Biexciton Binding Energy in Similar Nanocrystals

Table S1 presents a list of previously reported values of the BX binding energy of cesium lead halide perovskite NCs. It includes only results for $CsPbBr_3$ and $CsPbI_3$ NCs, as investigated in this work. We adopt the convention used in the main text, where attractive exciton-exciton interaction is regarded as positive BX binding energy. Inspection of the data presented in the table reveals a lack of consensus among the reported values. In addition, an objective comparison of the measurements is made difficult by variations in the size and confinement regime of the particles studied. The last row of the table contains the ensemble results from our present work.

S2 Synthesis Protocol

This section describes the synthesis protocol of the $CsPbBr_3$ and $CsPbI_3$ nanocrystals (NC) used in this work.

Materials. Cs_2CO_3 (99.995%, Sigma-Aldrich), octadecene (ODE, 90%, Sigma-Aldrich), oleic acid (OA, 90%, Sigma-Aldrich), oleylamine (OLA, 70%, Sigma-Aldrich), $PbBr_2$ (98%, Sigma-Aldrich), PbI_2 (99%, Aldrich), toluene (99.8%, Sigma-Aldrich, anhydrous), hexane (99.5%, Sigma-Aldrich, anhydrous) ammonium tetrafluoroborate (NH_4BF_4, 99.999%, Sigma-Aldrich), tetradecylphosphonic acid (TDPA, 99%, Sigma-Aldrich), Trioctylphosphine oxide (TOPO, 90%, Sigma-Aldrich)

Cs-Oleate Preparation. Cs_2CO_3 (101.7 mg), OA (312.5 µL) and ODE (5 mL) were mixed in a 50 mL round bottom flask, heated at 120 °C under vacuum for one hour. Then the temperature was raised to 160 °C and the mixture was kept for 10 min under Ar atmosphere. For the injection procedure, Cs-oleate was kept at 120 °C under Ar.

Synthesis of $CsPbBr_3$ Nanocrystals. $CsPbBr_3$ NCs were synthesized according to a reported recipe[11] with slight modifications. ODE (5 mL) and $PbBr_2$ (69 mg) were mixed in a 25 mL 3-neck flask and dried under vacuum for one hour at 120 °C. Then, under Ar atmosphere, dried OA (0.5 mL) and dried OLA (0.5 mL) were injected to the mixture. The temperature was raised to 180 °C and kept for 10 min. Cs-oleate solution (0.4 mL) was swiftly injected, and after 25 s the reaction mixture was cooled by ice water bath.

For the purification of the NCs, the crude solution was centrifuged at 6000 rpm for 5 min. After the centrifuge, the supernatant was discarded and the particles were re-

Table S1: **Measured values of the BX binding energy in cesium lead halide nanocrystals published in the literature.** $\langle N \rangle$ is the average number of photons absorbed per particle per pump pulse. Positive BX binding energy values correspond to an attractive exciton-exciton interaction.

Reference	Technique*	Material	Edge length (nm)	$\langle N \rangle$	BX binding energy (meV)
Wang *et al.* (2015) [1]	PDPL (CRYO)	$CsPbBr_3$	9	**	≈ 50
Makarov *et al.* (2016) [2]	TA (SD)	$CsPbI_3$	11.2 ± 0.7	0.1	11
Castaneda *et al.* (2016) [3]	TRPL	$CsPbBr_3$	≈ 7.4 ***<br>≈ 11.5 ***	≈ 2	≈ 100<br>≈ 30
		$CsPBI_3$	≈ 7.4 ***<br>≈ 12.8 ***		≈ 90<br>≈ 25
Aneesh *et al.* (2017) [4]	TA (SD)	$CsPbBr_3$	11	≈ 0.04	≈ 30
Yin *et al.* (2017) [5]	SP (CRYO)	$CsPbI_3$	≈ 9	≈ 0.05	14.26 ± 1.53
Yumoto *et al.* (2018) [6]	TA (SD)	$CsPbI_3$	6	0.1	≈ 35
Ashner *et al.* (2019) [7]	TA	$CsPbBr_3$	6<br>8<br>10	0.3	-10<br>-3<br>-2
Huang *et al.* (2020) [8]	2DES (CRYO)	$CsPbBr_3$	9	< 0.1	$25 - 40$
Shen *et al.* (2021) [9]	TA	$CsPbBr_3$	16	6.42<br>12.8	61.2<br>21.7
Dana *et al.* (2021) [10]	TA (SD)	$CsPbBr_3$	6 ± 0.7	≥ 4	~ -100
This work	HS	$CsPbBr_3$<br>$CsPbI_3$	5.9 ± 1.3<br>7.2 ± 1.9	≈ 0.1<br>≈ 0.3	10 ± 6<br>1 ± 9

* **PDPL** - Power dependent PL, **CRYO** - at cryogenic temperatures, **TA** - Transient absorption, **SD** - short delay, **TRPL** - Time resolved PL, **SP** - Single particle PL spectroscopy, **2DES** - Two-dimensional electron spectroscopy, **HS** - Heralded spectroscopy.
** No $\langle N \rangle$ quoted. Pump intensity varied from 4.5 to 54.7 μJ
*** Estimated from cross section data.

dispersed in anhydrous toluene forming colloidally stable solution.

The surface treatment of the colloidal $CsPbBr_3$ NCs was performed following the procedure reported in ref 12 with some modifications. Preparation of saturated NH_4BF_4 salt solution: toluene (2 mL, anhydrous) and NH_4BF_4 (10 mg) were stirred for 10 min, sonicated for 10 min and then centrifuged at 6000 rpm for 5 min. NH_4BF_4 salt precipitation was discarded, resulting in a saturated solution. NH_4BF_4 saturated solution (1 mL) was then stirred with $CsPbBr_3$ NCs precipitation in toluene (0.25mL) for 30 min, creating surface treated $CsPbBr_3$ NCs.

Synthesis of $CsPbI_3$ Nanocrystals. $CsPbI_3$ NCs were synthesized according to the recipe reported in ref 13 with minor modifications. ODE (5 mL), PbI_2 (86.7 mg), OLA (1 mL, anhydrous), TDPA (120 mg) and TOPO (1.47 mg) were mixed in a 50 mL 3-neck flask and dried under vacuum for one hour at 120 °C. The temperature was raised to 280 °C and kept for 10 min under Ar atmosphere. Then Cs-oleate solution (0.4 mL) was quickly injected, and after 15 s the reaction mixture was cooled by ice-water bath.

Purification procedure – crude solution was centrifuged at 6000 rpm for 5 min. Supernatant was discarded and precipitates were washed in anhydrous hexane, following additional centrifuge procedure (6000 rpm for 5 min).

S3 Supporting Analyses

This section describes the additional analyses performed on the collected data, on-top of the heralded spectroscopy. It describes the estimation of the average number of absorbed photons per excitation pulse ($\langle N \rangle$), the zero delay normalized second order correlation of photon arrival times ($g^{(2)}(0)$) and the *gated* zero delay normalized second order correlation of photon arrival times ($\hat{g}^{(2)}(0)$).

$\langle$N$\rangle$ Estimation. The average number of absorbed photons per excitation pulse, $\langle N \rangle$, was estimated from the ratio of detected BX–1X photon pairs to the total number of single detections. This ratio can be defined as the following:

$$\alpha \equiv \frac{N_2}{N_1} = \frac{p_{abs}(\geq 2) \cdot QY_{BX} \cdot QY_{1X} \cdot \eta \cdot {p_{det}}^2}{p_{abs}(\geq 1) \cdot QY_{1X} \cdot \eta \cdot p_{det}} = \frac{p_{abs}(\geq 2)}{p_{abs}(\geq 1)} \cdot QY_{BX} \cdot p_{det} \quad \text{(S1)}$$

N_2 is the number of detected photon pairs as described in the main text. N_1 is the number of single photons detected within a the 1X time-gate, *i.e.* during a time window of 0.5–30 ns following any excitation pulse (see section S4). $p_{abs}(k)$ is the probability a NC absorbs k photons in a single excitation pulse. QY_{BX} and QY_{1X} are the quantum yields of the BX and 1X, respectively. That is, the probability for the respective excited state to relax radiatively to the next lower energy state. η is a scalar factor accounting for single and pair detections filtered out due to the 1X temporal gate described above. p_{det} is the probability to detect a photon that was emitted from the NC. Note that the temporal gating of N_1 serves not only to cancel out the factor of η but also to filter out most contributions from the biexciton and trion states to the single-photon signal (see section S5).

$g^{(2)}(0)$, described in further detail in the next subsection, is:

$$g^{(2)}(0) = \frac{p_{abs}(\geq 2) \cdot QY_{BX} \cdot QY_{1X} \cdot {p_{det}}^2}{\frac{p_{abs}(\geq 1)^2}{2} \cdot {QY_{1X}}^2 \cdot {p_{det}}^2} = \frac{2 \cdot p_{abs}(\geq 2)}{p_{abs}(\geq 1)^2} \cdot \frac{QY_{BX}}{QY_{1X}} \quad \text{(S2)}$$

In the first line, the nominator is the probability to absorb, emit and detect two photons following the same excitation pulse. The denominator represents the probability to absorb, emit and detect a single photon in each of two separate excitation pulses.

Absorption statistics are expected to follow a Poissonian distribution. That is, the probability to absorb n photons in any single excitation pulse is:

$$p_{abs}(n) = \frac{\langle N \rangle^n}{n!} \cdot e^{-\langle N \rangle} \quad \text{(S3)}$$

and hence:

$$p_{abs}(\geq n) = 1 - \sum_{k=o}^{n-1} \frac{\langle N \rangle^k}{k!} \cdot e^{-\langle N \rangle} \quad \text{(S4)}$$

Plugging this into eq S2, we see that for $\langle N \rangle \ll 1$, the expression for $g^{(2)}(0)$ simplifies to the more commonly quoted expression: $g^{(2)}(0) \approx \frac{QY_{BX}}{QY_{1X}}$.

Finally, we can combine all the previous equations, to attain an expression for $\langle N \rangle$:

$$\langle N \rangle = -\ln\left(1 - \frac{2 \cdot \alpha}{QY_{1X} \cdot g^{(2)}(0) \cdot p_{det}}\right) \quad \text{(S5)}$$

α and $g^{(2)}(0)$ are measured quantities extracted from the same data used for the heralded spectroscopy. QY_{1X} was measured, for an ensemble of NCs, by an absolute photoluminescence (PL) quantum yield spectrometer (Quantaurus-QY, Hamamatsu), and is ∼100% for $CsPbBr_3$ and ∼42% for $CsPbI_3$. p_{det} was previously estimated for ∼2.01 eV emission and a different grating[14] as $p_{det} \approx 1.5 \times 10^{-2}$. According to the factory characterization of the grating and the measured spectral response of the detector, we can estimate $p_{det} \approx 2.5 \times 10^{-2}$ for $CsPbBr_3$ and $p_{det} \approx 1.2 \times 10^{-2}$ for $CsPbI_3$.

For the measurements shown in Figures 3 and 4 of the main text, $\langle N \rangle_{CsPbBr_3} = 0.13 \pm 0.04$ and $\langle N \rangle_{CsPbI_3} = 0.28 \pm 0.18$. For these $\langle N \rangle$ values, the probability to excite a NC more than twice is low ($p_{abs}(\geq 3) \ll p_{abs}(\geq 2)$). Combined with the typically lower quantum yields of higher multiexcitonic states (as evident in their shorter PL decay lifetimes[15]) we estimate that the contribution of triply and higher excited states to the heralded spectroscopy signal is negligible. We note that in both eq S1 and eq S2 we neglect the contribution of higher multiexcitonic states. Some of these contributions cancel out in eq S5, while others are negligible due to the low $\langle N \rangle$ and multiexciton quantum yield.

$\mathbf{g^{(2)}(0)}$ Calculation. $g^{(2)}(0)$ was calculated and corrected for errors arising from crosstalk and dark counts by the method described in ref 16. Briefly, we treat the SPAD array pixels as the arms of a multiple-port Hanbury Brown and Twiss photon correlation setup. A histogram of detection pairs by the delay between the detections (τ) is generated to extract the second order correlation of photon arrival times ($G^{(2)}(\tau)$). The number of detection pairs not originating in photon pairs (*i.e.* due to dark counts or inter-pixel crosstalk) is estimated from the measured intensity and SPAD array characterization, and subtracted from the histogram. Figure S1 shows the corrected $G^{(2)}(\tau)$ extracted from the same single-NC measurement featured in Figure 2 of the main text. It features a series of peaks separated by the pulse repetition rate (200 ns), and widened due to the finite PL decay lifetime (∼6 ns, see section S5). The zero delay peak is visibly attenuated compared to the other peaks, indicating photon antibunching (a lower probability to detect two photons following the same excitation pulse

as compared to detecting twice one photon following separate pulses). As described in the main text, this is due to the higher rate of non-radiative Auger processes in doubly-excited NCs, competing with radiative PL. The ratio between the area under the center peak, and the area under any other peak is termed the zero delay normalized second order correlation of photon arrival times, or $g^{(2)}(0)$. As described in the previous subsection, for the pump intensities used in this work, $g^{(2)}(0) \approx \frac{QY_{BX}}{QY_{1X}}$.

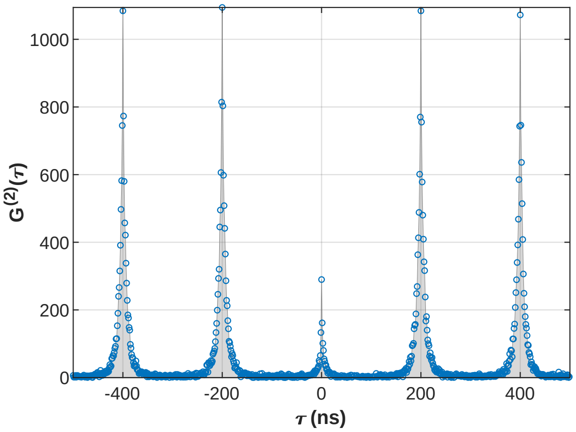

Figure S1: **Second order correlation of photon arrival time.** The second order correlation of photon arrival times for a single-NC measurement. The value of $G^{(2)}(\tau)$ represents the number of photon detection pairs with intra-detection delay of τ over the entire measurement. The attenuated zero delay peak is indicative of photon antibunching.

$\hat{\mathbf{g}}^{(2)}(\mathbf{0})$ Calculation. As described in the main text, $\hat{g}^{(2)}(0)$ is estimated by first post-selecting only detections within a time window of 1–30 ns after any excitation pulse, and then passing the filtered detections through the $g^{(2)}(0)$ analysis described in the previous subsection. The lower bound of the time-gate (1 ns) filters out multiexcitonic emission which features sub-ns PL decay lifetimes (see section S5). The upper bound (30 ns) serves to lower noise due to dark counts with minimal loss of 1X signal (as done in the heralded spectroscopy analysis, see section S4). Due to the complexity of crosstalk correction in this case we omit the center ±625 ps of each $G^{(2)}(\tau)$ peak. This delay time window for the zero delay peak accounts for 99.5% of crosstalk detection pairs.

S4 System Parameters

The experimental apparatus and analysis parameters are detailed in ref 14. The few modifications made to support the different PL parameters of the NCs used in this work are detailed in this section, and include: emission filters, spectrometer grating, instrument response function (IRF), temporal gating values and number of array pixels used.

Emission Filters. The fluorescence signal collected by the microscope was filtered by a dichroic beamsplitter (FF484-FDi02-t3, Semrock) and a longpass filter (BLP01-473R, Semrock).

Grating. The grating used in this work is a 333 g/mm plane ruled reflection grating, with 5.7° nominal blaze angle (53-*-321R, Richardson). This resulted in a reciprocal linear dispersion of 2.8×10^{-5} at the detector plane, and a spectral resolution of ∼4.5 Å. The detector active pixel pitch is 52.4 µm, and as a result, the pixel pitch in wavelength is ∼1.5 nm. This corresponds to pixel pitch of ∼7 meV and ∼4 meV at the emission spectral ranges of $CsPbBr_3$ and $CsPbI_3$, respectively.

Instrument Response Function. The only update to the detector from ref 14 is an updated firmware that enables significantly better performance of the time-to-digital converters (TDC), and consequently an improved IRF. The IRF, seen in Figure S2, is characterized by illuminating the detector directly with the synchronized excitation laser, and summing detections over 30 array pixels. The single-peak IRF features ∼180 ps full width at half maximum (FWHM). This response is a convolution of the excitation pulse temporal width and the timing jitter of the pixels (∼105 ps FWHM).

Analysis Parameters. Due to the improved IRF (see previous subsection), and shorter 1X fluorescence decay lifetime (see section S5), the temporal gates used to minimize the dark count rate (DCR) in ref 14 were refined: For the first photon of the pair (BX) only detections between −0.5 ns to 1 ns delay from the fluorescence temporal peak were considered. Pairs were post-selected such that the second detection (1X) is detected within 0.5 ns to 30 ns following the first. Both BX and 1X upper gates are at least a factor of 3 longer than the respective fluorescence decay lifetime (τ in section S5), and thus serve to lower the DCR with negligible loss of signal. The lower bound of the BX (−0.5 ns from the fluorescence temporal peak) ensures detections before the overall fluorescence temporal peak are not lost. The lower bound of the 1X (0.5 ns after the BX) ensures correct identification of arrival order (it's significantly larger than the IRF FWHM), and filters out most of the inter-pixel crosstalk, as it is characterized by similar timescales as the IRF.

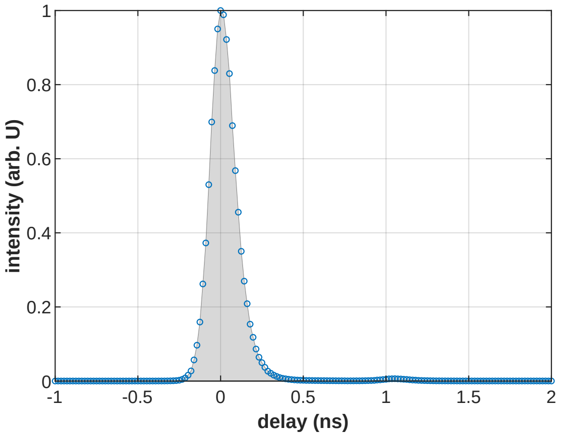

Figure S2: **Instrument response function.** The IRF, recorded by illuminating the SPAD array directly with the excitation laser. The presented histogram is generated according to the delay of each detection from the preceding excitation pulse, and summed over 30 detector pixels. Zero time delay is chosen as the maximal intensity delay-bin.

Number of Array Pixels Used. To minimize DCR, only a subset of the detector's 512 pixels was utilized. In $CsPbBr_3$ measurements, 30 pixels of the linear SPAD array were used, spanning the range of 2.32 meV to 2.53 meV photon energies. For $CsPbI_3$ measurements, 43 pixels of the array were used, spanning the range of 1.76 meV to 1.93 meV photon energies. One pixel in the 43 pixel range used for $CsPbI_3$ was malfunctioning and was hence omitted from the presented analyses.

S5 Photoluminescence Decay Lifetimes

This section describes the methods used to estimate the 1X PL decay lifetime and an upper bound on the BX PL decay lifetime from the collected data. The results support the analysis parameter choices detailed in section S4, and supply further reassurance to the identification of the 1X emission signal in heralded spectroscopy.

Single-Exciton. PL decay lifetime was estimated from a histogram of photon detections by their delay from the preceding excitation pulse. The blue trace in Figure S3 represents such a histogram for the single-NC measurement shown in Figure 2 of the main text. The purple trace represents a multiexponent fit of the form:

$$y = \sum_k a_k \cdot e^{-\frac{t}{\tau_k}} \tag{S6}$$

. For this measurement the fitted coefficients were: $\tau_{1,2,3,4} \approx 0.3, 1.6, 6.6, 36.6\,\text{ns}$ and $a_{1,2,3,4} \approx 0.69, 0.16, 0.20, 0.01$. The first two fast decay components have significant contribution only at the first ∼ns following the excitation pulse. We estimate that they account for some combination of PL from multi-excitonic states, PL from the charged trion state[14] and the IRF of our system (see section S4). The long decay τ_4 accounts for less than 1% of the signal. Finally, $\tau_3 \approx 6.6\,\text{ns}$ is the dominant component between 0.5 and 25 ns delay, and we assign it to the 1X PL decay lifetime. For the NCs in Figure 3 and 4 of the main text, 1X PL decay lifetimes are $5.9 \pm 1.6\,\text{ns}$ ($CsPbBr_3$) and $8.1 \pm 1.4\,\text{ns}$ ($CsPbI_3$).

Red bars in Figure S3 represent a histogram of the delay between BX and 1X detection for of all BX-1X photon pairs detected in the same measurement by heralded spectroscopy. To allow comparison, the right axis is scaled only by a scalar factor compared to the left axis. The good temporal agreement between the intra-pair delay (red bars) and the 1X dominated 0.5-30 ns emission (blue trace) further supports the designation of the second photon of the pair as 1X emission, and $\tau_3 \approx 6.6\,\text{ns}$ as its PL decay lifetime.

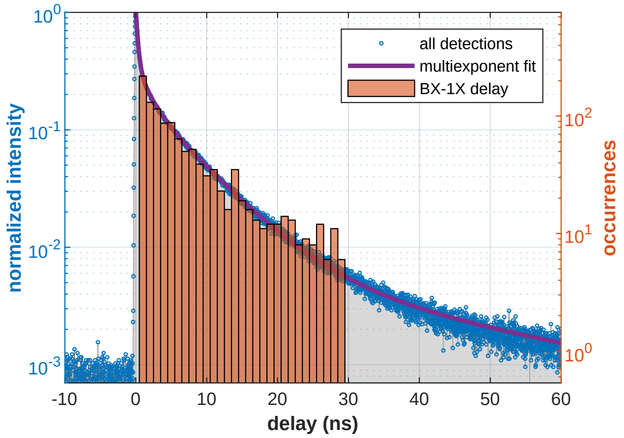

Figure S3: **Single-exciton photoluminescence decay lifetime.** Histogram of single-photon detection delays from the preceding excitation pulse, over a single-NC measurement (blue rings), and a fit to a multiexponential delay (purple line). Red bars are a histogram of delays between the two detections for all post-selected BX–1X pairs from the same measurement. To allow comparison, the right axis is scaled by a single scalar factor, such that the 1 ns delay bins of both histograms coincide.

Biexciton. An upper bound for the BX PL decay lifetime is estimated from the delays between the first detections in each post-selected BX–1X pair and the preceding excitation pulse. Figure S4 presents a histogram of such delays for the single-NC measurement featured in Figure 2 of the main text. Evidently, the distribution is a convolution of the IRF (Figure S2) and the BX PL decay lifetime. To set an upper bound on the BX PL decay lifetime, we fit an single-exponent decay distribution to all positive BX delays, using a maximum

likelihood estimate (red line, zero time delay is chosen as the delay with maximum single-photon detections). For this specific NC the result is $\tau \approx 190$ ps. For the NCs featured in Figure 3 and 4 of the main text, the estimated upper bounds on BX PL decay lifetimes are 234 ± 44 ns ($CsPbBr_3$) and 306 ± 50 ps ($CsPbI_3$). Indeed, previously reported values lie within these bounds.[3,7,15]

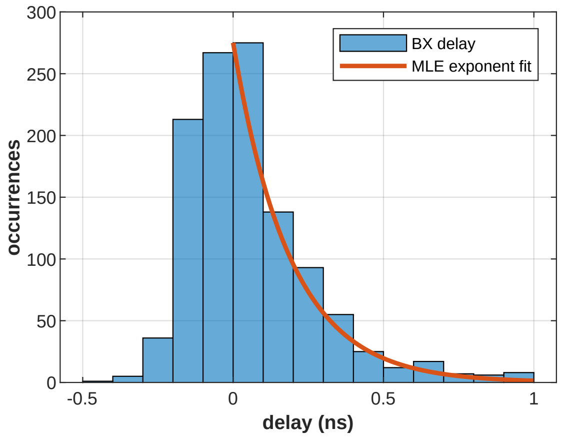

Figure S4: **Biexciton photoluminescence decay lifetime.** Histogram of BX detections' delay from the excitation pulse (blue bars). The observed temporal shape implies a convolution of the IRF (Figure S2) and the BX PL decay lifetime. Red line represents a single-exponent fit using a maximum likelihood estimation on all detections with positive delay ($\tau \approx 190$ ps).

The exact values of PL decay lifetime have no consequence for the validity of the heralded measurements, and are given here as an additional insight extracted from the same data-set. The approximate values and bounds, however, are used to justify the temporal-gating of BX and 1X detections in the heralded spectroscopy method (section S4) and gated $\hat{g}^{(2)}(0)$ (section S3).

S6 Biexciton Spectrum Broadening

Figure S5a and b present histograms of the BX and 1X emission spectra width for each NC featured in Figures 3 and 4, respectively, of the main text. The values presented are the full width at half maximum (FWHM) as extracted from the same Cauchy-Lorentz fits used to estimate the BX and 1X emission peaks in the main text. Figure S5c shows the distribution of BX to 1X spectra broadening ratio, calculated as the ratio between the BX and 1X emission spectra FWHM for each NC. As seen in Figure S5a and c, for $CsPbBr_3$ NCs, the BX spectrum is typically broader than the 1X by a ratio of 1.4 ± 0.4. The BX to 1X spectrum broadening ratio distribution for $CsPbI_3$ is 1.2 ± 0.7. However, the results for $CsPbI_3$ NCs are less conclusive, due the lower signal and smaller statistics compared to $CsPbBr_3$ NCs.

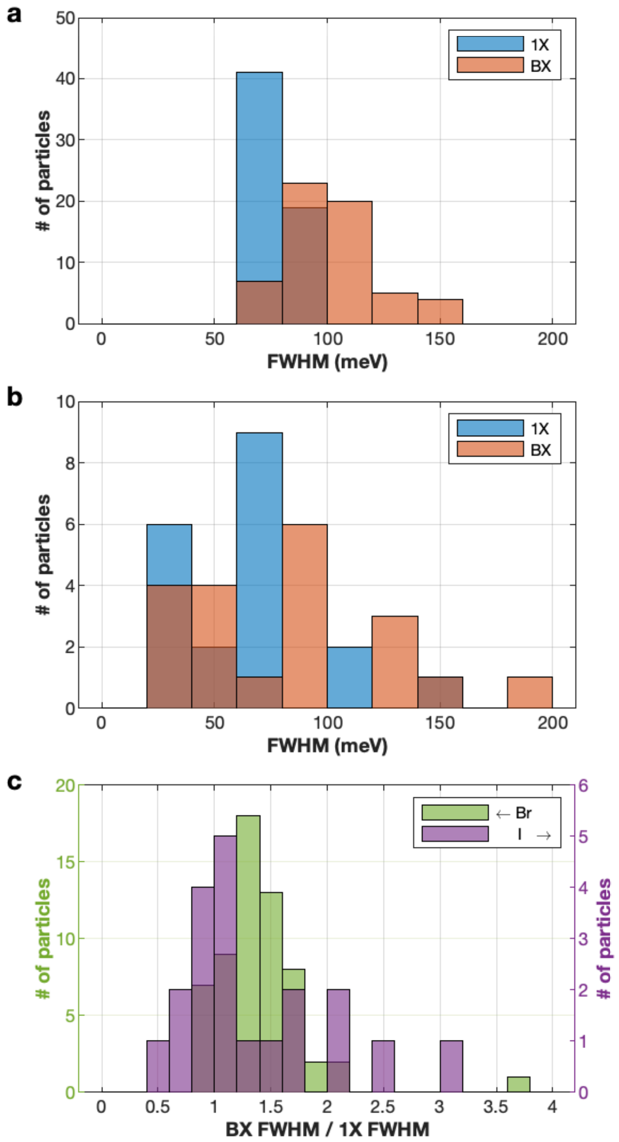

Figure S5: **BX spectrum broadening.** Histograms of BX and 1X spectra FWHM (extracted from the Cauchy-Lorentz fit described in the main text) for the $CsPbBr_3$ **(a)** and $CsPbI_3$ **(b)** NCs featured in Figures 3 and 4, respectively, of the main text. **c)** Histogram of the BX to 1X spectrum width ratio recorded for each NC, for $CsPbBr_3$ (green) and $CsPbI_3$ (purple).

References

(1) Wang, Y.; Li, X.; Song, J.; Xiao, L.; Zeng, H.; Sun, H. All-Inorganic Colloidal Perovskite Quantum Dots: A New Class of Lasing Materials with Favorable Characteristics. *Adv. Mater.* **2015**, *27*, 7101–7108.

(2) Makarov, N. S.; Guo, S.; Isaienko, O.; Liu, W.; Robel, I.; Klimov, V. I. Spectral and Dynamical

Properties of Single Excitons, Biexcitons, and Trions in Cesium-Lead-Halide Perovskite Quantum Dots. *Nano Lett.* **2016**, *16*, 2349–2362.

(3) Castañeda, J. A.; Nagamine, G.; Yassitepe, E.; Bonato, L. G.; Voznyy, O.; Hoogland, S.; Nogueira, A. F.; Sargent, E. H.; Cruz, C. H.; Padilha, L. A. Efficient Biexciton Interaction in Perovskite Quantum Dots under Weak and Strong Confinement. *ACS Nano* **2016**, *10*, 8603–8609.

(4) Aneesh, J.; Swarnkar, A.; Kumar Ravi, V.; Sharma, R.; Nag, A.; Adarsh, K. V. Ultrafast Exciton Dynamics in Colloidal CsPbBr3 Perovskite Nanocrystals: Biexciton Effect and Auger Recombination. *J. Phys. Chem. C* **2017**, *121*, 4734–4739.

(5) Yin, C.; Chen, L.; Song, N.; Lv, Y.; Hu, F.; Sun, C.; Yu, W. W.; Zhang, C.; Wang, X.; Zhang, Y.; Xiao, M. Bright-Exciton Fine-Structure Splittings in Single Perovskite Nanocrystals. *Phys. Rev. Lett.* **2017**, *119*, 026401.

(6) Yumoto, G.; Tahara, H.; Kawawaki, T.; Saruyama, M.; Sato, R.; Teranishi, T.; Kanemitsu, Y. Hot Biexciton Effect on Optical Gain in CsPbI3 Perovskite Nanocrystals. *J. Phys. Chem. Lett.* **2018**, *9*, 2222–2228.

(7) Ashner, M. N.; Shulenberger, K. E.; Krieg, F.; Powers, E. R.; Kovalenko, M. V.; Bawendi, M. G.; Tisdale, W. A. Size-Dependent Biexciton Spectrum in CsPbBr3 Perovskite Nanocrystals. *ACS Energy Lett.* **2019**, *4*, 2639–2645.

(8) Huang, X.; Chen, L.; Zhang, C.; Qin, Z.; Yu, B.; Wang, X.; Xiao, M. Inhomogeneous Biexciton Binding in Perovskite Semiconductor Nanocrystals Measured with Two-Dimensional Spectroscopy. *J. Phys. Chem. Lett.* **2020**, *11*, 10173–10181.

(9) An, L.; Pan, K.; Shen, X.; Wang, S.; Geng, C.; Li, L.; Zhao, E.; Sun, J.; Wu, W. Red Shift of Bleaching Signals in Femtosecond Transient Absorption Spectra of CsPbX3(X = Cl/Br, Br, Br/I) Nanocrystals Induced by the Biexciton Effect. *J. Phys. Chem. C* **2021**, *125*, 5278–5287.

(10) Dana, J.; Binyamin, T.; Etgar, L.; Ruhman, S. Unusually Strong Biexciton Repulsion Detected in Quantum Confined CsPbBr3 Nanocrystals with Two and Three Pulse Femtosecond Spectroscopy. *ACS Nano* **2021**, *15*, 23.

(11) Cao, Y.; Zhu, W.; Li, L.; Zhang, Z.; Chen, Z.; Lin, Y.; Zhu, J. J. Size-Selected and Surface-Passivated CsPbBr3 Perovskite Nanocrystals for Self-Enhanced Electrochemiluminescence in Aqueous Media. *Nanoscale* **2020**, *12*, 7321–7329.

(12) Ahmed, T.; Seth, S.; Samanta, A. Boosting the Photoluminescence of CsPbX3 (X = Cl, Br, I) Perovskite Nanocrystals Covering a Wide Wavelength Range by Postsynthetic Treatment with Tetrafluoroborate Salts. *Chem. Mater.* **2018**, *30*, 3633–3637.

(13) Pan, L.; Zhang, L.; Qi, Y.; Conkle, K.; Han, F.; Zhu, X.; Box, D.; Shahbazyan, T. V.; Dai, Q. Stable CsPbI3 Nanocrystals Modified by Tetra-*n*-Butylammonium Iodide for Light-Emitting Diodes. *ACS Appl. Nano Mater.* **2020**, *3*, 9260–9267.

(14) Lubin, G.; Tenne, R.; Ulku, A. C.; Antolovic, I. M.; Burri, S.; Karg, S.; Yallapragada, V. J.; Bruschini, C.; Charbon, E.; Oron, D. Heralded Spectroscopy Reveals Exciton-Exciton Correlations in Single Colloidal Quantum Dots. *Nano Lett.* **2021**, *21*, 6756–6763.

(15) De Jong, E. M.; Yamashita, G.; Gomez, L.; Ashida, M.; Fujiwara, Y.; Gregorkiewicz, T. Multiexciton Lifetime in All-Inorganic CsPbBr3 Perovskite Nanocrystals. *J. Phys. Chem. C* **2017**, *121*, 1941–1947.

(16) Lubin, G.; Tenne, R.; Antolovic, I. M.; Charbon, E.; Bruschini, C.; Oron, D. Quantum Correlation Measurement with Single Photon Avalanche Diode Arrays. **2019**, *27*, 32863–32882.
